# What are the neurodevelopmental outcomes of children with asymptomatic congenital cytomegalovirus infection at birth? A systematic literature review

**DOI:** 10.1002/rmv.2555

**Published:** 2024-06-21

**Authors:** Angeliki Smyrli, Vishnuga Raveendran, Simone Walter, Waheeda Pagarkar, Nigel Field, Seilesh Kadambari, Hermione Lyall, Heather Bailey

**Affiliations:** ^1^ Institute for Global Health University College London London UK; ^2^ St George's University Hospitals NHS Foundation Trust London UK; ^3^ Department of Audiological Medicine Great Ormond Street Hospital for Children NHS Foundation Trust London UK; ^4^ Department of Paediatric Infectious Diseases and Immunology Great Ormond Street Hospital London UK; ^5^ University College London Great Ormond Street Institute of Child Health London UK; ^6^ Imperial College Healthcare NHS Trust London UK

**Keywords:** asymptomatic, congenital cytomegalovirus, neurodevelopment

## Abstract

Congenital cytomegalovirus (cCMV) is among the most common congenital infections globally. Of 85%–90% cCMV‐infected infants without symptoms at birth, 10%–15% develop sequelae, most commonly sensorineural hearing loss (SNHL); their childhood neurodevelopmental outcomes are less well understood. Embase and MEDLINE were searched for publications from 16^th^ September 2016 to 9th February 2024 to identify studies reporting primary data on neurodevelopmental outcomes in children with asymptomatic cCMV (AcCMV), measured using assessment tools or as evaluated by the study investigators, clinicians, educators, or parents. The Newcastle‐Ottawa scale was applied to studies to assess risk of bias. Of 28 studies from 18 mostly high‐income countries, there were 5‐109 children with AcCMV per study and 6/28 had a mean or median age at last follow‐up of ≥5 years. Children with AcCMV had better neurodevelopmental outcomes than children with symptomatic cCMV in 16/19 studies. Of 9/28 studies comparing AcCMV with CMV‐uninfected children, six reported similar outcomes whilst three reported differences limited to measures of full‐scale intelligence and receptive vocabulary among children with AcCMV and SNHL, or more generally in motor impairment. Common limitations of studies for our question were a lack of cCMV‐uninfected controls, heterogeneous definitions of AcCMV, lack of focus on neurodevelopment, selection bias and inadequate follow‐up. There was little evidence of children with AcCMV having worse neurodevelopmental outcomes than CMV‐uninfected children, but this conclusion is limited by study characteristics and quality; findings highlight the need for well‐designed and standardised approaches to investigate long‐term sequelae.

**Abbreviations**
AcCMV: asymptomatic cCMV
ADHD: attention deficit hyperactivity disorder
ASD: autistic spectrum disorder
cCMV: congenital cytomegalovirus
DBS: dried blood spot
DQ: development quotient
NOS: Newcastle‐Ottawa scale
ScCMV: symptomatic cCMV
SNHL: sensorineural hearing loss

## 1 INTRODUCTION

**FIGURE 1:**
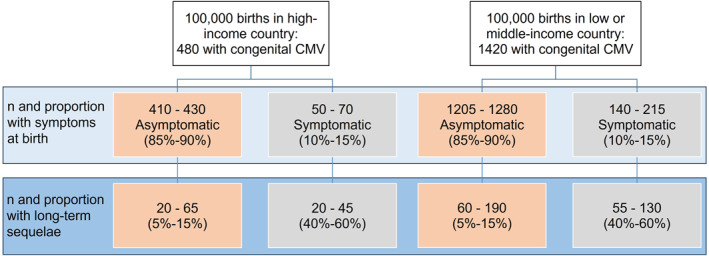
Estimated number of children with long‐term sequelae associated with congenital CMV infection, by presence of clinically detectable symptoms at birth, per 100,000 births in high income and low or middle‐income countries. Estimates of cCMV prevalence from^1^ and estimates of proportions with symptoms at birth and long‐term sequelae from.^3^ Numbers rounded to nearest 5.

Prevalence at birth of congenitally acquired CMV (cCMV) is estimated at 0.48% (95% CI 0.40%–0.59%) in high‐income countries and 1.42% (0.97%–2.08%) in low and middle‐income countries, where maternal seroprevalence is higher, making this among the most frequent congenital infections globally.^1^, ^2^ At birth, around 10%–15% of cCMV‐infected newborns have clinically detectable symptoms of cCMV, of whom an estimated 40%–58% experience persistent sequelae including sensorineural hearing loss (SNHL), ophthalmological outcomes, and neurodevelopmental delay.^3^ For those identified as symptomatic at birth, trials have shown a modest benefit of antiviral treatment started in the first 4 weeks of life for hearing and developmental outcomes.^4^, ^5^, ^6^ Children with asymptomatic cCMV (AcCMV) at birth have a lower risk of long‐term sequelae (estimated to occur in 10%–15%^3^) but account for the majority of children with cCMV‐related sequelae of any severity overall, owing to this group's much larger size (Figure 1). Most infants without symptoms at birth are likely to remain undiagnosed in the absence of universal screening. SNHL is the most commonly reported sequela attributed to cCMV infection,^7^ with permanent childhood hearing impairment reported in at least 40% of children with symptomatic cCMV (ScCMV) at birth, and up to 30% in children with AcCMV at follow‐up between 6 months and 18 years, in a recent review.^8^

For children with cCMV but without apparent symptoms at birth (i.e. 85%–90%), the implications of cCMV for outcomes other than hearing, such as neurodevelopmental outcomes, are less clear. Early studies in which children with cCMV were systematically identified through screening and prospectively followed up to at least 3 years of age showed that for those without symptoms of cCMV, neurodevelopment was similar to uninfected controls.^9^, ^10^ A systematic review conducted in 2016 by Bartlett et al.^11^ found that of 11 studies published between 1974 and 2016 comparing neurodevelopmental outcomes in children who had AcCMV at birth versus uninfected control groups, eight studies reported no difference at follow‐up age between 10 months and 12.5 years. Of the remaining three studies, differences were based on outcomes of a single child, did not persist over time or were among children in whom hearing loss had not been excluded as a potential confounder of the association between AcCMV and poorer verbal Intelligence Quotient (IQ).^11^ However, only five of these 11 studies had follow‐up to at least four years of age (439 controls and 397 AcCMV total across the five studies), and methodological heterogeneity limited comparisons between (and possibly within) studies and generalisability of findings to a contemporary population. With the introduction of newborn hearing screening, children with hearing loss in the absence of other apparent symptoms in the newborn period are now increasingly included in the ‘symptomatic at birth’ group, although this categorisation is applied inconsistently,^11^ which also poses challenges in comparing other, related neurodevelopmental outcomes (e.g. speech and language development) across groups and studies.

A complete picture of the implications of cCMV for the longer term health and development of children without symptoms at birth is needed to inform future research and policy, including around screening of newborns for cCMV and potential treatment and vaccination strategies. We systematically reviewed literature published since the last review by Bartlett et al.^11^ on neurodevelopmental outcomes of children with AcCMV.

## 2 METHODS

This review was conducted in line with the PRISMA, 2020 statement^12^ and registered in the International Prospective Register of Systematic Reviews (PROSPERO; registration number: CRD42022331535^13^). Our methodology is adapted from an earlier review by Bartlett et al.^11^ where acknowledged. Embase and MEDLINE databases were searched for articles published from 16 September 2016 to 6 May 2022, subsequently updated to 9 February 2024, to capture papers published since the earlier review. The full search strategy can be found at: https://www.crd.york.ac.uk/PROSPEROFILES/331535_STRATEGY_20240313.pdf.^13^

Two reviewers independently screened article titles and abstracts and, for articles not excluded, the full text. Data extraction was independently validated by two reviewers, with conflicts discussed and resolved by a third reviewer.

### 2.1 Definitions

cCMV infection was defined as a positive CMV result diagnosed through viral isolation by culture and/or identification of viral DNA through polymerase chain reaction in neonatal saliva and/or urine and/or blood sample taken within the first 21 days of life, while CMV‐uninfected was defined as a negative result on this same category of sample.^14^

We considered as ‘asymptomatic’ (hereafter referred to as ‘AcCMV’), a child with a positive CMV result but without clinically detectable symptoms of CMV at birth, according to the definition used in each included study. Unlike the earlier review,^11^ we did not exclude studies that categorised children with abnormalities detected through further investigations (e.g. neuroimaging) as ‘asymptomatic’, because we are interested in the outcomes of all children who, in the absence of newborn CMV screening, may not be diagnosed.

Neurodevelopmental impairment was taken as defined by the individual studies, so included any below average performance on developmental, neurological, or cognitive assessment tools (or using stricter cut‐offs), or as evaluated by the study investigators, clinicians, educators, or parents.

### 2.2 Eligibility criteria

Studies included were quantitative observational studies with follow‐up data beyond 30 days of life on neurodevelopment outcomes (neurological, speech and language, cognitive or motor) among children with AcCMV at birth. We did not include studies of children with neurodevelopmental delay who had been retrospectively tested for cCMV unless a comparison group without the outcome had also been included (i.e. to allow the neurodevelopmental delay attributable to cCMV to be estimated). Case reports and case series were excluded, as were studies with outcomes reported for <3 children with AcCMV, non‐primary studies and those not published in English. Conference abstracts were included only when linked to a full‐text article. For multiple publications from the same cohort, only the one with the largest population was included, unless different papers contained information on different outcomes, in which case this is indicated in the results. Studies reporting solely on other outcomes (e.g. hearing, vestibular dysfunction) or not disaggregating outcomes for children with AcCMV were excluded. Our eligibility criteria differed from Bartlett et al^11^ in places: we included studies in which some children categorised as AcCMV had received antivirals (rather than making this part of our exclusion criteria) and extracted data on antiviral use; we placed a minimum restriction on follow‐up duration (30 days or more); we did not exclude retrospective cohort studies of children with neurodevelopmental delay, provided other criteria were met, and we used a broader definition of AcCMV (see definitions section).

Information extracted from studies included study design, characteristics and population, reason for CMV testing (e.g., screening and/or diagnostic investigation), the CMV diagnostic tests used, antiviral treatment received, method(s) for assessing neurodevelopmental outcomes, definitions used for ScCMV (and therefore AcCMV), and for neurodevelopmental impairment, duration of follow‐up/age at assessments, and results for neurodevelopmental outcomes. Neurodevelopmental outcomes were stratified by hearing status where available.

### 2.3 Assessing neurodevelopment and follow‐up

Early child development is defined by the WHO as the period from conception to 8 years,^15^ marked by gross motor abilities, fine‐motor coordination, and development of language abilities.^16^ Assessments to identify neurodevelopmental delays have limitations at the youngest ages (floor effects) and problems may be more likely to be identified at older ages, including school entry.^16^ Neurodevelopmental outcomes can be measured using a variety of methods including validated assessment tools (sometimes requiring administration by a professional), or evaluations by clinicians, parents or educators. Outcome measures are related to the type of study and population—for example, serial measures using assessment tools are likely to only be available in prospective studies or in children with a clinical indication for follow‐up.

A post‐hoc decision was made to conduct statistical tests (Fisher's exact or chi‐squared tests) to compare outcomes in AcCMV and cCMV‐uninfected groups, where an included publication reported sufficient data but with no statistical test.

### 2.4 Risk of bias assessment

The Newcastle‐Ottawa Scale (NOS) for assessing the quality of non‐randomised studies was used to assess risk of bias in the studies, including selection bias in the study population and in measurement of exposure and outcome, bias in the availability of follow‐up information, adequacy of follow‐up duration and comparability of the AcCMV and comparison groups where applicable.^17^ For the purposes of this review, follow‐up to at least age 5 years was scored as ‘adequate’ and defined as a mean or median follow‐up period of at least 5 years or assessments scheduled to at least 5 years within each study.

## 3 RESULTS

**FIGURE 2:**
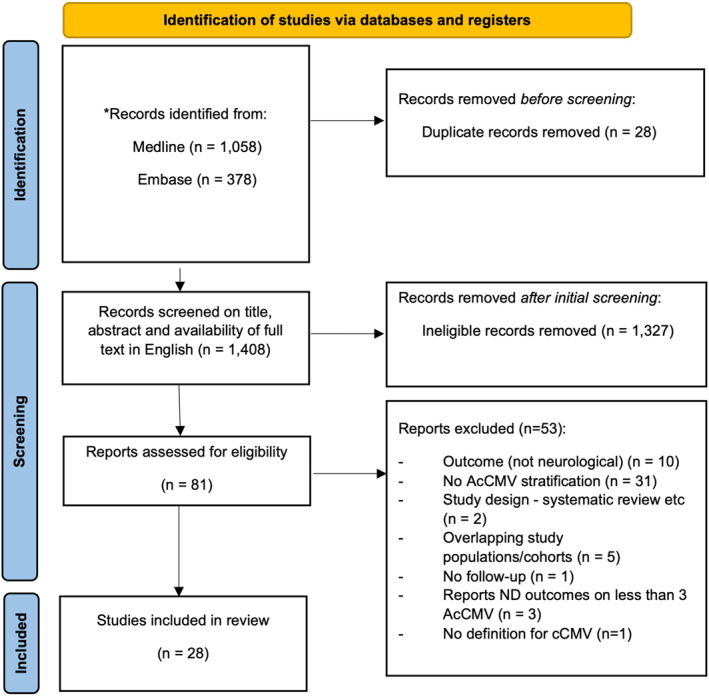
PRISMA flow diagram detailing search and selection process applied during systematic review, using template.^12^ * = Of this total, 1118 (807 from Medline and 311 from Embase) were retrieved using the original search dates up to 6th May 2022, with an additional 318 (251 from Medline and 67 from Embase) retrieved when searches were updated to 9th February 2024.

Of 1436 articles published between September 2016 and February 2024, 1408 remained after de‐duplication and 81 articles underwent full‐text assessment; after agreement between two reviewers, 28 met inclusion criteria (Figure 2).

### 3.1 Study characteristics

**FIGURE 3:**
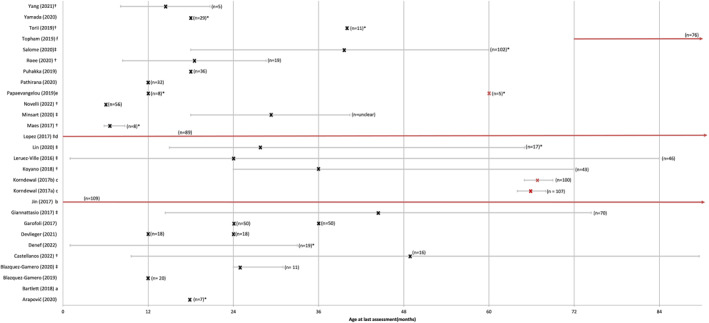
Length of follow‐up of study participants presented as mean, median or scheduled age at last assessment (in months) for each study included in the systematic review. x = Age at which results were obtained (predetermined assessment point or through active monitoring), bars indicate range of ages at last assessment, † = Mean age at last assessment, ‡ = Median age at last assessment, *n* = number of AcCMV with follow‐up, * = follow‐up length reported for AcCMV group only (whereas others are length of follow‐up for all study participants with follow‐up), Red ‘x’ or ranges: Studies with follow‐up of mean/median or scheduled assessment of 5 or more years. ^a^Bartlett et al.: Unclear follow‐up duration but minimum 30 days after birth for AcCMV group (number of AcCMV with FU = 79). ^b^Jin et al.: Longitudinal study was up to 18 years but no clear indication of age at neurodevelopmental assessment (arrow indicates that duration of follow‐up exceeds *x* axis). ^c^Korndewal et al.: Data retrospectively collected and represent cumulative total of impairments diagnosed up to the first 6 years of life (rather than diagnosed/present at 6 years of age). ^d^Lopez et al.: Median age at last assessment was 17 years for all tests and groups, apart from expressive vocabulary which was 13 years (arrow indicates that duration of follow‐up exceeds x axis). ^e^Papaevangelou et al.: 8 children assessed at 12 months of age and 5 of these were assessed at 5 years of age (12 months and 5 years follow‐up plotted as a ‘x’). ^f^Topham et al.: Outcome was assessed at minimum one point between the ages of 18 years of age (indicated on plot with arrow from 72 months onwards).

Table 1 shows the characteristics of 28 studies included in this review. These include prospective (*n* = 20),^18^, ^20^, ^21^, ^24^, ^25^, ^26^, ^27^, ^28^, ^30^, ^32^, ^33^, ^34^, ^36^, ^37^, ^38^, ^39^, ^40^, ^41^, ^42^, ^45^ retrospective (*n* = 7)^22^, ^23^, ^29^, ^31^, ^35^, ^43^, ^44^ and cross‐sectional studies (*n* = 1),^19^ conducted in 18 different countries; by region, 16 studies were conducted in Europe,^18^, ^20^, ^21^, ^22^, ^23^, ^24^, ^25^, ^26^, ^28^, ^30^, ^32^, ^34^, ^36^, ^43^, ^44^, ^45^ five in East Asia,^27^, ^29^, ^37^, ^38^, ^39^ four in North America^31^, ^40^, ^41^, ^42^ and one each in Australia,^19^ South Africa^33^ and Israel.^35^ Enrolment of study participants began after 2010 in 11 studies,^18^, ^20^, ^21^, ^23^, ^29^, ^32^, ^33^, ^34^, ^35^, ^37^, ^39^ between 2000 and 2010 in 13 studies^22^, ^24^, ^25^, ^26^, ^27^, ^28^, ^30^, ^31^, ^36^, ^38^, ^43^, ^44^, ^45^ and before 2000 for 4 studies.^19^, ^40^, ^41^, ^42^ Study populations (including, for some studies, the total number screened for CMV) varied in size, ranging between 36 and 207,547 newborns/infants/children; the number of children with AcCMV and follow‐up was between 5 and 109 children per study, total: 1218 (953 unique individuals after removing double‐counting from studies reporting on overlapping cohorts). Figure 3 shows the age at which neurodevelopmental outcomes were ascertained. The age at last follow‐up ranged between 1 month and 18 years, with 6/28 studies (from three cohorts) including populations with at least 5 years of follow‐up^40^, ^41^, ^42^, ^43^, ^44^, ^45^ (duration unclear for one study).^19^ cCMV was identified among children in the included studies via population screening (*n* = 13),^18^, ^21^, ^27^, ^29^, ^33^, ^34^, ^38^, ^39^, ^41^, ^42^, ^43^, ^44^, ^45^ diagnostic testing of populations with suspected infection or clinical indications (*n* = 4),^19^, ^20^, ^22^, ^26^ testing of infants born to women with primary CMV infection during pregnancy (*n* = 5)^23^, ^24^, ^32^, ^35^, ^37^ or a combination of these (*n* = 6).^25^, ^28^, ^30^, ^31^, ^36^, ^40^ Four studies only included infants born to women with primary maternal CMV infection,^23^, ^24^, ^32^, ^35^ whereas eight studies reported the proportion of children born to women with primary and non‐primary maternal CMV infections during pregnancy,^21^, ^26^, ^28^, ^30^, ^31^, ^33^, ^34^, ^45^ although not all reported neurodevelopmental outcomes by these groups. The majority of studies did not report type or timing of maternal infection (*n* = 16).^18^, ^19^, ^20^, ^22^, ^25^, ^27^, ^29^, ^36^, ^37^, ^38^, ^39^, ^40^, ^41^, ^42^, ^43^, ^44^

**TABLE 1:** Study characteristics of the 28 studies included in the systematic review.

First author (year of publication)	Country	Study design	Study period	Study population	Study population with follow‐up data
Studies with less than 5 years of follow‐up					
^18^Arapović (2020)	Bosnia and Herzegovina	Prospective cohort	Infants born: November 2015—October 2016, then followed up	‐ Screening as part of the study ‐ 1293 neonates tested for cCMV ‐ 8/1293 were cCMV‐positive neonates (all AcCMV) ‐ 7/8 with FU and 1/8 without FU information	7 AcCMV
^19^Bartlett (2018)	Australia	Cross‐sectional surveillance	January 1999 to December 2016	‐ Surveillance of new cCMV cases seen by participating clinicians ‐ 302/509 of cCMV‐positive cases reported to Australian paediatric surveillance unit were included in study ‐ 88/302 AcCMV and 214/302 ScCMV (1 AcCMV and 57 ScCMV treated with antivirals) ‐ 79/88 AcCMV born 2004 onwards with reported developmental and hearing outcomes	79 AcCMV
^20^Blazquez‐Gamero (2019)	Spain	Prospective cohort	January 2011 to May 2017	‐ CMV testing for neonatal clinical indication; all had neuroimaging 107/421 cCMV‐positive neonates included in study (73/77 ScCMV and 23/30 AcCMV treated with antivirals) ‐ 71 cCMV‐positive with FU	20 AcCMV and 51 ScCMV
^21^Blazquez‐Gamero (2020)	Spain	Prospective cohort	Enrolment: February 2017 to February 2018, then followed up	‐ Neonatal screening ‐ 3226/4097 neonates eligible for study (weekend births ineligible) ‐ 3190/3226 had parental consent ‐ 15/3190 were cCMV‐positive neonates ‐ Maternal infection data available for 14/15; 10/14 were nonprimary infections and 4/14 were primary infections ‐ 13/15 cCMV‐positive cases with FU (6 AcCMV and 2 ScCMV treated with antivirals), 2 AcCMV lost to FU	11 AcCMV and 2 ScCMV
^22^Castellanos (2022)	Spain	Retrospective, observational	January 2003 to August 2017	‐ CMV testing for neonatal clinical indication; all had neuroimaging ‐ 36 cCMV‐positive with FU included in study and under paediatric neurology FU (20 patients treated with antivirals)	16 AcCMV and 20 ScCMV
^23^Denef (2022)	Belgium	Retrospective cohort	January 2011 and October 2018	‐ Newborn testing in response to evidence of maternal primary CMV infection ‐ 90/135 pregnant women with primary CMV infection eligible for study ‐ 35/91 fetuses were cCMV‐positive ‐ 12/35 TOPs; of 23 cCMV‐positive infants, 20 were AcCMV and 3 were ScCMV ‐ 19/20 AcCMV and all 3 ScCMV with FU (2 ScCMV treated with antivirals)	19 AcCMV and 3 ScCMV
^24^Devlieger (2021)	Austria, Belgium, Germany, Hungary, and Italy	Observational follow‐up of children born to pregnant women enroled in a prospective randomised open‐label phase 3 study	Pregnant women enroled from October 2008; child followed up (up to 24 m) with last neonate study visit in December 2016	‐ Newborns tested due to evidence of primary CMV infection in mother during pregnancy (identified through randomised open‐label phase III study of cytomegalovirus‐specific hyperimmunoglobulin) ‐ 9535/15,474 of pregnant women screened had negative CMV serology and met eligibility for study ‐ 94 babies were born to women who seroconverted (of the 9535 screened) and subsequently included for evaluation ‐ 16/45 babies with confirmed cCMV were born to women in the treatment group and 13/28 to women in control group (after exclusions due to off label treatment and babies without infection status available) ‐ Of the 29 cCMV‐infected children (of whom 3 were treated with antivirals), 2 had missing symptom status at birth, 4 were ScCMV and 23 were AcCMV. There were also 44 cCMV‐uninfected children ‐ 22/27 cCMV‐infected children with symptom status at birth and 28/44 cCMV‐uninfected were tested for at least one neurodevelopmental anomaly at minimum 12 months	18 AcCMV, 4 ScCMV and 28 controls
^25^Garofoli (2017)	Italy	Prospective cohort	Neonates born at or referred to centre: 2007–2012, then followed up	‐ Diagnostic testing of neonates born at or before transferred to the infection centre ‐ 70 cCMV‐positive cases ‐ 50 AcCMV and 20 ScCMV (17 ScCMV treated with antivirals) with FU	50 AcCMV and 20 ScCMV
^26^Giannattasio (2017)	Italy	Prospective cohort	Neonates born: 2002–2015, then followed up	‐ CMV testing for neonatal and/or maternal clinical indication ‐ 158/224 cCMV‐positive infants eligible for study ‐ 93/158 children and 65/158 children born to mothers with a primary and a nonprimary CMV infection, respectively ‐ 70 AcCMV and 88 ScCMV (51 ScCMV treated with antivirals) with FU	70 AcCMV and 88 ScCMV
^27^Koyano (2018)	Japan	Prospective cohort	2006–2010	‐ Neonatal screening for CMV ‐ 72/23,405 were cCMV‐positive of screened neonates ‐ 54 AcCMV and 17 ScCMV, 1 did not meet inclusion criteria for study ‐ 60 patients with FU (10 ScCMV treated with antivirals)	43 AcCMV and 17 ScCMV
^28^Leruez‐Ville (2016)	France	Prospective cohort	2008–2013	‐ Testing in response to prenatal fetal diagnosis (from amniocentesis following documented maternal seroconversion or suggestive ultrasound features) ‐ 82/92 diagnosed fetuses with complete CMV status ‐ 82 fetuses: 24 TOP and 58 cCMV‐positive neonates (81/82 born to mothers with a primary and 1/82 to a nonprimary CMV infection) ‐ 58 cCMV‐positive cases at birth: 47 AcCMV (38 with no US features; 9 with non‐severe features) and 11 ScCMV (1 had severe and 10 non‐severe US findings in utero) ‐ 46/47 AcCMV and all 11 ScCMV had FU	46 AcCMV and 11 ScCMV
^29^Lin (2020)	Japan	Retrospective, observational	Neonates born: April 2010—September 2017	‐ Data collected in medical claims database ‐ 53/207,547 were cCMV‐positive neonates in observation period ‐ All 53 cCMV‐positive had FU (10 ScCMV and 4 AcCMV treated with antivirals)	17 AcCMV and 36 ScCMV
^30^Maes (2017)	Belgium	Prospective cohort (Flemish CMV registry)	Registry set up in January 2007	‐ cCMV‐positive groups identified through testing of infants born to women with known CMV infection during pregnancy or with symptoms ‐ 40 participants: 8 AcCMV, 8 ScCMV without SNHL, 8 ScCMV with SNHL, 8 with connexin mutation (isolated hearing‐impaired controls) and 8 typically developing controls with normal hearing ‐ 7/8 AcCMV, 7/8 ScCMV with normal hearing and 7/8 ScCMV with hearing loss were born to women with reported trimester of seroconversion during pregnancy ‐ 24/40 included in analysis for this review (excluded 8 with connexin mutation and 8 controls as they were selected not to have outcomes of interest) (10 ScCMV treated with antivirals: 6 ScCMV without SNHL, 4 ScCMV with SNHL)	8 AcCMV, 8 ScCMV without SNHL and 8 ScCMV with SNHL
^31^Minsart (2020)	Canada	Retrospective cohort	Referred between 2003 and 2017	‐ CMV testing for neonatal and/or maternal clinical indication ‐ 84 cCMV‐positive fetuses were eligible for study of the 133 mother‐child pairs ‐ Maternal CMV infection available for 59 fetuses, of which 16 were primary, 26 likely primary, 9 non‐primary and 8 with unknown maternal CMV infections type ‐ 77 live births (51 patients treated with antivirals); 18 AcCMV, 3 AcCMV with SNHL, 47 moderate‐severe ScCMV and 9 mild ScCMV ‐ 74 survived neonatal period	74 neonates survived the neonatal period
^32^Novelli (2022)	Italy	Prospective cohort	Enrolment: 2013–2020	‐ Identified due to primary CMV infection diagnosed in mother during pregnancy, all born at term (≥37 weeks), with neurodevelopmental follow‐up and assessment as part of standard of care ‐ 56 AcCMV in total; of which 54 had neurological follow‐up at 6 months and 56 had Bayley‐III scales scores	56 AcCMV
^33^Pathirana (2020)	South Africa	Prospective cohort	Screened: May–December 2016, then followed up	‐ Screening as part of study ‐ 130/2,685 screened neonates enroled in study ‐ 123/130 (94.6%) of cases and controls were born to women with non‐primary CMV infection, type of maternal CMV infection is undetermined for 7/130 (5.3%) ‐ 46/130 infants cCMV‐positive and 84/130 cCMV‐uninfected infants ‐ 35/46 cCMV‐positive and 74/84 cCMV‐uninfected matched controls completed ND assessments at 12 months FU (1 ScCMV and 2 AcCMV treated with antivirals)	32 AcCMV, 3 ScCMV and 74 controls
^34^Puhakka (2019)	Finland	Prospective cohort	Neonates born: September 2012–January 2015, then followed up	‐ Screening as part of the study ‐ 56/19,868 of screened infants had positive saliva CMV‐PCR at birth ‐ 40/56 infants had confirmed cCMV infection and were matched with 54 eligible cCMV‐uninfected controls ‐ Of 40 cCMV‐positive, 20/40 had nonprimary, 18/40 had primary maternal CMV infection and type of maternal CMV infection was undetermined in 2/40 infants ‐ 88/94 infants had neurological FU	36 AcCMV, 1 ScCMV and 51 controls
^35^Roee (2020)	Israel	Retrospective cohort	July 2015–March 2018	‐ Testing of an at‐risk group born to women with primary CMV infection ‐ 149 pregnant mothers referred ‐ 27/149 fetuses diagnosed with subtle findings on fetal brain imaging, forming study cohort ‐ 19/27 cCMV‐positive (all AcCMV; 4 were treated with antivirals) and 8/27 cCMV‐uninfected with FU	19 AcCMV and 8 controls
^36^Salome (2020)	Italy	Prospective cohort	2002–2018	‐ Testing of an at‐risk group born to women with evidence of CMV seroconversion or neonates born at their centre with clinical indication ‐ 258/326 neonates tested/referred were cCMV‐positive ‐ 125/258 were AcCMV and 133/258 were ScCMV ‐ 23/125 of the AcCMV group had FU less than 12 months (therefore excluded from study) and FU of 133 ScCMV not reported ‐ 102/125 AcCMV had more than 1 year FU	102 AcCMV
^37^Torii (2019)	Japan	Prospective cohort	April 2014–February 2017	‐ Testing of an at‐risk group born to mothers with serological evidence of CMV infection in pregnancy ‐ 11,753 pregnant women CMV screened; 685 neonates born to borderline IgM‐positive mothers or IgG‐seroconverted mothers, were subsequently tested for cCMV via PCR ‐ 11/685 tested neonates were cCMV‐positive (all asymptomatic)	11 AcCMV
^38^Yamada (2020)	Japan	Prospective cohort	Born: November 2009 and March 2018, then followed up	‐ Neonatal screening for CMV ‐ 56/11,768 diagnosed as cCMV‐positive neonates ‐ 29/33 AcCMV and 19/23 ScCMV (all 19 ScCMV with FU treated with antivirals) had neurological FU	29 AcCMV and 19 ScCMV
^39^Yang (2021)	Taiwan	Prospective cohort	Enrolment: May 2016 to Dec 2018 and then followed up	‐ Neonatal screening for CMV ‐ 1532/3273 had consent to undergo CMV screening ‐ 7/1532 were cCMV‐positive and 1525/1532 were cCMV‐uninfected (all cCMV‐uninfected only have hearing data presented) ‐ 5/7 cCMV‐positive had FU (all AcCMV), 2/7 lost to FU (unknown reason)	5 AcCMV
Studies with 5 or more years of follow‐up					
^40^Jin (2017), ^41^Lopez (2017) and ^42^Topham (2019)^a^	USA	Prospective cohort—Three papers with overlapping populations but different outcome measures	Children born in 1982–1992 and then followed up	‐ Screening as part of study or referred for clinical indication ‐ Jin (2017): 237 infants and children enroled for study from CMV screening of 32,543 neonates/referred from other centres. 186 cCMV‐positive and 51 cCMV‐uninfected with FU ‐ Lopez (2017): 135/32,543 neonates were cCMV‐positive and enroled with 42 unmatched cCMV‐uninfected controls born within 6 days of an infant who tested positive for CMV 92/135 AcCMV and 43/135 ScCMV 89/92 AcCMV and 40/42 controls had ND assessment, therefore included in analysis ‐ Topham (2019): 109 AcCMV, 77 ScCMV and 51 cCMV‐uninfected controls enroled; 37 of total lost to FU before age 6 141/200 completed at least 1 ND assessment	Jin (2017): 109 AcCMV, 77 ScCMV and 51 controls
					Lopez (2017): 89 AcCMV and 40 controls
					Topham (2019): 76 AcCMV, 36 ScCMV and 29 controls
^43^, ^44^Korndewal (2017a and 2017b papers)^a^	Netherlands	Retrospective cohort—two papers with overlapping populations but different outcome measures	CMV retrospectively diagnosed between November 2012‐June 2014, for children born in 2008	‐ Newborn dried blood spots testing for CMV (screening) ‐ 32,486/73,693 had parental consent for testing; of which 31,484 had DBS tested for cCMV ‐ 154/31,484 were cCMV‐positive and 2 tested cCMV‐positive elsewhere (subsequently included in cohort) ‐ Korndewal (2017a): 133 cCMV‐positive (107 AcCMV and 26 ScCMV) and 274 CMV‐uninfected infants had consent for data retrieval on outcomes (1 patient treated with antivirals) ‐ Korndewal (2017b): 123/133 cCMV‐positive (100 AcCMV and 23 ScCMV) and 242/274 CMV‐uninfected infants with developmental delay results	Korndewal (2017a): 107 AcCMV, 26 ScCMV and 274 controls
					Korndewal (2017b): 100 AcCMV, 23 ScCMV and 242 controls
^45^Papaevangelou (2019)	Greece	Prospective cohort	Neonates born: 2008–2010, then followed up	‐ Newborn screening as part of study ‐ 2149 neonates enroled in study ‐ 10/2149 were cCMV‐positive neonates (all asymptomatic at birth) ‐ 9/10 neonates were born to mothers with nonprimary CMV infection and 1/10 was born to a mother with primary CMV infection ‐ 8/10 cCMV‐positive had FU	8 AcCMV

Abbreviations: AcCMV, children with asymptomatic congenital cytomegalovirus; cCMV, congenital cytomegalovirus; FU, follow‐up; ND, neurodevelopment (al); ScCMV, children with symptomatic congenital cytomegalovirus; SNHL, sensorineural hearing loss; TOPs, Termination of pregnancy; US, ultrasound.

^a^ Studies with overlapping populations, therefore combined in Table 1. The study populations are disaggregated in cells accordingly.

The definitions of AcCMV and ScCMV varied across studies (Table 2), with 19 studies including hearing loss^19^, ^20^, ^21^, ^22^, ^23^, ^25^, ^26^, ^27^, ^28^, ^29^, ^30^, ^32^, ^36^, ^37^, ^38^, ^39^, ^43^, ^44^, ^45^ and six studies excluding children with isolated hearing loss in their definition of ScCMV,^24^, ^31^, ^33^, ^40^, ^41^, ^42^ whilst three did not state how these children were categorised.^18^, ^34^, ^35^ Additionally, 17 studies included^19^, ^20^, ^21^, ^23^, ^25^, ^26^, ^27^, ^28^, ^30^, ^31^, ^32^, ^36^, ^37^, ^38^, ^43^, ^44^, ^45^ and six excluded^22^, ^24^, ^33^, ^40^, ^41^, ^42^ abnormal brain imaging results in their definition of ScCMV, and five studies did not specify or were unclear about this aspect of their definition^18^, ^29^, ^34^, ^35^, ^39^(Table 2). Many studies used urine or saliva samples to diagnose cCMV infection, reflecting their prospective designs; three studies retrospectively tested children using stored neonatal dried blood spots (DBS)^43^, ^44^, ^45^ and two studies included a combination of children who were either retrospectively or prospectively tested.^19^, ^22^ Neurodevelopmental outcomes were predominantly assessed by the use of instruments such as Bayley Scales of Infant and Toddler Development, Denver Scale, Griffiths Mental Developmental Scales, the Peabody Developmental Motor Scales, Wechsler Intelligence Scale for Children, among others (*n* = 16)^22^, ^24^, ^25^, ^26^, ^27^, ^30^, ^31^, ^32^, ^33^, ^34^, ^36^, ^38^, ^41^, ^42^, ^43^, ^45^ (Table 2). Others included neurological and developmental examinations by paediatricians (*n* = 3)^18^, ^20^, ^21^ or self‐reporting of symptomology and milestones by newborns/child's parent or their school (*n* = 2).^35^, ^44^ Seven studies reported neurodevelopmental delay outcomes without defining how they were assessed.^19^, ^23^, ^28^, ^29^, ^37^, ^39^, ^40^ Antivirals were received by children with AcCMV in six studies^19^, ^20^, ^21^, ^29^, ^33^, ^35^ and by children with ScCMV in 11 studies.^19^, ^20^, ^21^, ^23^, ^25^, ^26^, ^27^, ^29^, ^30^, ^33^, ^38^ Four studies reported use of antivirals in children with cCMV without disaggregating this information by infant's symptom status,^22^, ^24^, ^31^, ^43^ while 12 studies did not report on antiviral use.

**TABLE 2:** Outcome assessment, length of follow‐up and neurodevelopmental outcomes of 28 studies included.

First author (year of publication)	Outcome assessment	Length of follow‐up	Study results	Risk of bias scoring (NOS)	Included in author's definition of symptomatic cCMV?	
					Hearing loss^†^	Brain imaging abnormalities^†^
Studies with less than 5 years of follow‐up						
^18^Arapović (2020)	Examination undertaken by paediatric neurologist and audiologic evaluations.	Evaluated at 18 months of age	7 AcCMV had no evidence of neurodevelopmental delay or hearing loss at FU.	3^a^	NS	NS
^19^Bartlett (2018)	Not specified	Unclear follow‐up duration but minimum 30 days after birth	1/79 AcCMV had developmental delay. 21/79 AcCMV developed hearing loss (overlap with developmental delay not reported).	2^a^	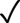	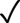
^20^Blazquez‐Gamero (2019)	Physical examination by paediatrician and/or a neuropaediatric assessed for neurologic abnormalities (including neurodevelopmental delay and motor impairment)	Follow‐up of 12 months	1/20 AcCMV had neurological abnormalities, none had SNHL. 15/51 ScCMV had neurological abnormalities (for 3/15 this was in combination with SNHL), and 10 ScCMV had SNHL only.	3	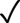	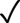
^21^Blazquez‐Gamero (2020)	Evaluation and/or examination assessment by neuropsychologist	Median: 25 months (IQR: 24–31)	1/11 AcCMV had language development delay with normal hearing (at 36 months)—This child was born to a woman with primary infection during pregnancy. 1/2 ScCMV had mild neurodevelopmental delay (mother had nonprimary infection). None of the 13 cCMV‐positive cases developed hearing loss during FU.	5	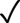	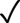
^22^Castellanos (2022)	Paediatric neurologist assessment and identification of following: Microcephalus, psychomotor impairment according to the Denver scale, motor alterations, behavioural alterations, cognitive alterations, and speech impairment.	Mean age: 49.6 months SD 40.0	3/16 AcCMV and 15/20 ScCMV had neurological sequalae. 12/36 of cCMV‐positive had hearing alterations (overlap with neurodevelopment or stratification by AcCMV/ScCMV not reported).	4	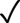	X
^23^Denef (2022)	Not specified	Followed for a range of 1–33 months for AcCMV; up to 56 months for ScCMV	17/19 AcCMV had normal neurodevelopmental outcomes at FU, two had non‐neurodevelopmental symptoms: One child presented with right‐sided facial paralysis at 3 months (resolved by 20 months), and one had hearing loss (at 5 months). 2/3 ScCMV had normal neurodevelopmental outcomes at FU and 1/3 had ASD and speech delay at 25 months.	4	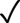	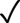
^24^Devlieger (2021)	Bayley cognitive/Motor scale at 12 and 24 months	Up to 24 months of FU, minimum: 12 months	1/18 AcCMV had Bayley motor anomalies reported at 12 months follow‐up (no hearing loss), 1/18 AcCMV had Bayley motor anomalies reported at 24 months (no hearing loss) and no clinical neurological or cognitive anomalies were reported among those with data available at 24 months. None of the 4 ScCMV had abnormalities detected at follow‐up (12 months for 2, 24 months for 2). 2/28 cCMV‐uninfected controls had motor abnormalities at 12 months follow up (Bayley) but none at 24 months (follow‐up available for 23 cCMV‐uninfected). No cognitive anomalies or clinical neurological anomalies reported for cCMV‐uninfected at 24 months among those with follow up available (*n* = 23).	3	X	X
^25^Garofoli (2017)	Griffiths' developmental scale (aged 18 and 24 months), Autism diagnostic observation schedule, Autism diagnostic interview (ADI‐R) and Vineland adaptive Behaviour scale	Results reported for 2 and 3 years of age	3/50 AcCMV and 1/20 ScCMV had suspected ASD at 24 months and two (2 AcCMV at birth) had ASD confirmed at 3 years.	4	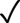	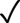
^26^Giannattasio (2017)	Griffiths mental developmental Scales, Denver test, Weschler scale (aged ≥30 months), and child behaviour checklist (CBCL) (aged ≥18 months). Ages of posture‐motor control milestone acquisition recorded.	Ages at last observation: Mean 3.8 years SD 2.6 (group 1—maternal primary infection) and mean 3.6 years SD 2.4 (group 2—maternal nonprimary infection) years old	6/70 AcCMV (all born to mothers with primary maternal infection) had neurodevelopmental impairments (2 mild motor impairments, 2 language impairments, 1 epilepsy case and 1 case of behavioural/emotional problems); 6/70 AcCMV (3 infants born to mothers with primary and 3 infants born to mothers with nonprimary maternal infection) had SNHL impairment (overlap with neurodevelopmental impairment not reported). 32/88 ScCMV (16 infants born to mothers with primary and 16 infants to mothers with nonprimary maternal infection) had neurodevelopmental impairments; 35/88 ScCMV had SNHL impairment (21 infants born to mothers with primary maternal infection and 14 infants born to mothers with nonprimary maternal infection).	4	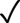	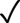
^27^Koyano (2018)	Wechsler intelligence scale for children (WISC‐III or IV) or Kyoto scale (for >2 years), and developmental quotient or intelligence quotient scoring	Minimum 2 year of follow‐up, average: 3 years and maximum: 6 years	5/43 AcCMV developed late‐onset sequelae: 2/43 had speech delay without hearing impairment aged 3 or above, 1/43 had late‐onset bilateral hearing impairment aged 1, 1/43 had ASD aged 3 and 1/43 had attention deficit hyperactivity disorder (ADHD) aged 6. 14/17 ScCMV had sequelae at follow‐up; of the 3 without sequelae apparent at aged 2 years, all were in the group of 10 children who had received antivirals.	5	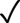	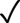
^28^Leruez‐Ville (2016)	Not specified	Median:24 months (range 1–84 months)	None of the 46 AcCMV developed neurological impairment or bilateral hearing loss at median 24 months follow‐up. 1/11 ScCMV developed motor and cognitive delay, 1/11 ScCMV was severely disabled (unspecified), 8/11 ScCMV developed unilateral hearing loss and 2/11 ScCMV had bilateral hearing loss (overlap between neurodevelopment and hearing loss not reported).	4	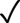	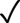
^29^Lin (2020)	Investigated occurrence of CMV‐related sequalae but no detail on how neurodevelopmental outcomes is measured	Median: 28 (range: 15–65) months for AcCMV and median: 35 (range: 8–88) months for ScCMV	2/17 AcCMV had developmental problems during FU, both had motor problems, one had speech problems and one had intellectual disability. Among AcCMV, there were two cases of SNHL (unclear whether these were the same individuals as those with developmental problems). 14/36 ScCMV had developmental problems (7 with speech problems, 4 with motor problems and 4 with intellectual disability; overlap not reported). Among ScCMV, there were 24 cases of SHNL, 7 with seizures/epilepsy and 6 with cerebral palsy.	5	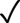	Unclear = brain abnormalities mentioned—But no mentioning of imaging specifically
^30^Maes (2017)	The peabody developmental motor scales (second edition)	Mean age: 6.8 months (range: 5.8–8.7 months) for AcCMV and ScCMV (range: 5.4–8.9 months)	No significant differences were obtained for the fine motor performance between any of the any groups (AcCMV vs. ScCMV with hearing‐impairment vs. ScCMV without hearing impairment). The ScCMV hearing‐impaired group presented lower gross motor performance when compared to the AcCMV group (*p* = 0.034).	3	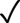	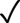
^31^Minsart (2020)	Bayley‐III, peabody developmental motor scale, Alberta infant motor scale, Conners scale, Wechsler intelligence scale IV and preschool and primary scale of intelligence, age‐and‐stages questionnaire 3, Denver Developmental screening test, and Kaufman‐ABC‐II.	Median: 29.5 months (IQR: 18.0–40.4).	Of total 74 surviving the neonatal period (a combination of AcCMV + those with mild symptoms or isolated SNHL + moderate‐severe ScCMV; exact breakdown not available), all 9 children with severe neurodevelopmental symptoms were in the moderate‐severe ScCMV group. None of the asymptomatic neonates, or neonates with mild symptoms developed severe outcomes, although 8 of these had isolated bilateral SNHL at FU.	4	X	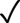
^32^Novelli (2022)	Neurological evaluation including assessment of motor and sensory function, primitive reflexes and tone. The Bayley‐III scales (cognitive, language and motor scales administered by clinician)	Follow‐up assessment at mean 6 months SD 0.67	Neurological examination was normal in 24/54 (44%) AcCMV children, slightly abnormal (minimal asymmetries, minimal alteration of movements, tone and/or posture) in 24/54 (44%) and mildly abnormal (mild defects of posture, movements and/or tone not affecting function) in 6/54 (11%); none had moderately or severely abnormal neurological status. Bayley‐III scale scores were lower than 1SD (<85) in the motor scale composite score for 8/54 (14%) children. No children had a score <85 in the cognitive or language domains (for 56 and 55 children, respectively). There was no statistically significant difference in either outcome by trimester of maternal infection. All had normal audiologic evaluation in first 6 months.	3^a^	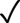	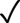
^33^Pathirana (2020)	The Bayley III scales of infant and toddler development administered	Follow‐up assessment to 12 months of age	At 6 months, 1/3 ScCMV born with microcephaly had neurodevelopmental delay. At 12 months, 1/32 AcCMV had motor delay, 1/3 ScCMV born with microcephaly had delay across all three domains (cognitive, language and motor) and 3/74 cCMV‐uninfected had a neurodevelopmental delay (two had language delay and one had motor delay) (comparing CMV‐positive and controls: OR 1.09, 95% CI 0.04–27.84, *p* = 0.958). At 12 months, 31/34 cCMV‐positive and 58/74 cCMV‐uninfected who complete hearing assessments had ‘normal’ assessment results.	5	X	X
^34^Puhakka (2019)	Griffiths mental development scales	Follow‐up assessments at 18 months	Griffiths mental development score was obtained at FU for 37 cCMV‐infected (36 AcCMV and 1 ScCMV) and 51 cCMV‐uninfected controls. The outcomes at 18 months did not differ significantly between the children with cCMV and cCMV‐uninfected controls (using χ2 or Fisher exact test: general quotient, *p* = 0.557; all subscales, *p* = 0.173–0.721). Hearing function also did not differ between cCMV‐infected and cCMV‐uninfected children at 18 months (4/54 cCMV‐infected infant ears and 6/80 cCMV‐uninfected infant ears failed Transient evoked otoacoustic emission testing, *p* = 1.0).	6	NS	NS
^35^Roee (2020)	Developmental assessment performed by telephone questionnaire conducted with the parents, including questions on any abnormal findings on routine assessments, or any relevant referrals or neurologic diagnoses made at paediatric neurodevelopment clinic.	Mean:18.5 months SD 10.1 months	All children (19 AcCMV and 8 cCMV‐uninfected) had FU and normal development reported, except one child with AcCMV who was diagnosed with unilateral hearing loss.	3	NS	NS
^36^Salome (2020)	Neurological examinations, psychomotor development test for first 3 months, psychomotor development test and clinical/lab evaluations (unclear whether this includes neurological evaluations) every 6 months up until 2 years	Age at last observation of AcCMV mean 3.3 years SD 1.8	None of the AcCMV (*n* = 102) developed later sequelae such as neurologic disorders or stable hearing loss during the FU but 14 children had fluctuating hearing impairment.	4^a^	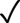	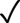
^37^Torii (2019)	Not specified	Mean age of last regular visit for 11 AcCMV was 40 months	None of the 11 AcCMV showed signs of developmental delay or SNHL at the last visit	4^a^	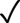	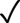
^38^Yamada (2020)	Neurodevelopmental outcomes including development quotient (measured using Kyoto scale of psychological development) and checking for hearing dysfunction, blindness, and epilepsy	Outcomes assessed at 18 months corrected age	1/29 AcCMV developed unilateral hearing loss and autism. 8/19 ScCMV had impairment according to DQ and 6 of those also had hearing loss; an additional 4 children with ScCMV and normal DQ had hearing loss.	5	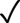	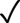
^39^Yang (2021)	Neurologic assessment (not specified)	Mean: 14.4 months SD 6.3	None of the five AcCMV developed hearing or other symptoms during FU.	2^a^	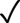	NS
Studies with 5 or more years of follow‐up						
^40^Jin (2017) (cohort overlaps with Lopez 2017 and Topham 2019)	Serial age‐appropriate neurodevelopmental evaluations (not specified)	Longitudinal study was 18 years but unclear timing of neurodevelopmental assessment	4/109 AcCMV, 24/77 ScCMV and 1/51 cCMV‐uninfected controls had neurodevelopmental delay (using χ2: *p* < 0.001 for comparison between symptomatic and asymptomatic, *p* < 0.001 for symptomatic vs. cCMV‐uninfected controls, and *p* = 1.0 for asymptomatic vs. cCMV‐uninfected controls). 19/109 AcCMV, 53/77 ScCMV and 1/51 cCMV‐uninfected controls had SNHL (overlap with neurodevelopmental delay outcomes not reported).	3	X	X
^41^Lopez (2017) (cohort overlaps with Jin 2017 and Topham 2019)	IQ score combined from: Wechsler intelligence scale for children (WISC) (third edition) and the Wechsler abbreviated scale of intelligence (WASI) at ages 6–18 years, Bayley scales of infant development (mental scale score) and McCarthy scales of Children's abilities (general cognitive index score). In addition: Peabody picture vocabulary test, expressive one word picture test, Woodcock‐Johnson tests of achievement (broad math and broad reading scores)	Median age at last assessment was 17 years for all tests and groups, apart from expressive vocabulary which was 13 years. Comparisons were made at 5 and 18 years for measures that increased linearly with age, plus 12 years for those that increased non‐linearly	Children were grouped for analysis according to their hearing at age 2 years: 78 AcCMV with normal hearing, 11 AcCMV with SNHL, 40 cCMV‐uninfected controls with normal hearing. Of note, 9/78 AcCMV with normal hearing and 3/40 cCMV‐uninfected controls were diagnosed with SNHL during FU. There was no statistically significant difference in any of the measures between the AcCMV group with normal hearing and the cCMV‐uninfected group. AcCMV with SNHL diagnosed by 2 years scored lower on the measure of full‐scale intelligence (by 7.0 points (SE = 0.3)), and on the measure of receptive vocabulary (by 13.1 points (SE 4.2)) than the cCMV‐uninfected control group (*p* < 0.05) but had similar scores across all other measures.	7	X	X
^42^Topham (2019) (cohort overlaps with Jin 2017 and Lopez 2017)	The behaviour assessment system for children (BASC‐2) (for inattention and hyperactivity), Wechsler intelligence scale for children (for IQ), Wechsler abbreviated scale of intelligence, the nonverbal scale of the Kaufman assessment battery for children, or the Leiter international performance scale– Revised (for children with sensorineural hearing loss)	Outcomes assessed at minimum one point between 6 and 18 years	Mean IQ scores for ScCMV were 19 points lower than AcCMV. 5/56 of ScCMV had an IQ below 70, however no cases among AcCMV or cCMV‐uninfected controls. There were no significant differences in the proportion of children with attention problems T‐scores ever ≥65 (17% vs. 24%; *p* = 0.407) or hyperactivity T‐scores ever ≥65 (20% vs. 14%; *p* = 0.490) among AcCMV versus cCMV‐uninfected controls. The proportion of children with attention problems T‐scores was significantly higher in the ScCMV versus the AcCMV group (39% vs. 17%; *p* = 0.012) but not versus the control group (39% vs. 24%; *p* = 0.221)—statistical test: χ^2^. Hearing loss was reported in 21% of AcCMV, 78% of ScCMV, and in 10% of cCMV‐uninfected (overlap with neurodevelopmental delay not reported).	4	X	X
^43^Korndewal (2017a) (cohort overlaps with Korndewal 2017b)	Neurological impairment, speech, and language development, and cognitive or motor developmental delay obtained from medical specialists' reports. Results from movement assessment battery for children (MABC) and the schlichting test for language production and Reynell test for language comprehension were used if recorded.	Retrospective study based on data collected of children from birth up to age 6 years mean age: 5 years 6 months SD: 2 months	19.6% of children with cCMV and 12.4% of those without cCMV presented symptoms potentially related to cCMV in the neonatal period. Neurological impairment was reported in 2.8% of AcCMV, 19.2% of ScCMV, 5.1% of uninfected. Cognitive impairment was reported in 3.7% of AcCMV, 15.4% of ScCMV, 1.1% of uninfected. Motor impairment was reported in 10.3% of AcCMV, 19.2% of ScCMV, 1.5% of uninfected. Speech‐language impairment was reported in 12.1% of AcCMV, 34.6% of ScCMV, 7.3% of uninfected. Risk differences presented for cCMV‐uninfected versus cCMV‐infected (combined AcCMV and ScCMV) only: these were statistically significant for cognitive, motor and speech‐language impairment, but not for neurological impairment. SNHL was reported in 2.8% of children with AcCMV, 7.7% with ScCMV and none of the cCMV‐uninfected group (overlap with other impairments not reported).	7	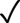	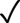
^44^Korndewal (2017b) (cohort overlaps with Korndewal 2017a)	Data collected from school and health‐care providers involved in care of children. Questionnaires sent to parents. The child development inventory (CDI) in Dutch was used to assess the development of the children	Retrospective study based on 5 years of school and healthcare data, coupled with parental questionnaire sent when child was aged 5 years, mean age: 5.6 SD: 0.2 years	CDI results show that overall proportions of children with each delay were similar in the cCMV versus CMV‐uninfected groups for language comprehension, fine and gross motor skills, self‐help, social, letters and numbers. For expressive language and general development, children with cCMV were more likely to have a delay than those uninfected, but the proportion reporting these delays among AcCMV was lower than in ScCMV (11% in AcCMV, 26.1% in ScCMV and 5.8% in uninfected for expressive language; 8% for AcCMV, 21.7% for ScCMV and 3.7% in uninfected for general development). Special needs education was attended by 5.3% in cCMV positive and 2.9% in cCMV‐uninfected. 4% and 5% of AcCMV and cCMV‐uninfected children had poor school results respectively. 3 AcCMV and 2 ScCMV were diagnosed with SNHL before age 5 or 6 years (overlap with neurodevelopmental delay not reported).	7	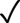	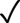
^45^Papaevangelou (2019)	Not specified for 12 months, Griffiths mental development scale for 5 years	8 cases had follow‐up for 12 months and 5 of these were assessed at 5 years of age	All 8 AcCMV had normal neurodevelopment and hearing at 12 months FU. The 5 AcCMV with 5 years FU had normal psychomotor development, receptive and expressive language skills, 2 of which had bilateral SNHL.	3^a^	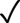	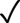

Abbreviations: ×, excluded in definition of symptomatic; ✓, included in definition of symptomatic (^†^i.e. could include children with isolated hearing loss or brain abnormalities); AcCMV, children with asymptomatic congenital cytomegalovirus; ASD, autism spectrum disorder; CI, confidence internal; CMV, congenital cytomegalovirus; DQ, development quotient; FU, follow‐up; HL, hearing loss; IQ, Intelligence Quotient; IQR, inter‐quartile range; NS, not specified—definition of symptomatic/asymptomatic was not defined clearly in the study; OR, odds ratio; PCR, polymerase chain reaction; ScCMV, children with symptomatic congenital cytomegalovirus; SD, standard deviation; SE, standard error; SNHL, sensorineural hearing loss.

^a^ In risk of bias scoring = no comparison group available, therefore denominator was 5 instead of 7, with a score of 7/7 meaning minimal bias in the Newcastle‐Ottawa Scale tool.

### 3.2 Neurodevelopmental outcomes in studies with less than 5 years of follow‐up

Table 2 summarises reported neurodevelopmental outcomes. Of 22 studies with follow‐up to less than five years, eight reported normal neurodevelopmental outcomes/development in all children with AcCMV at last study follow‐up, at an average of between 12 and 40 months of age.^18^, ^23^, ^28^, ^31^, ^35^, ^36^, ^37^, ^39^ The AcCMV children were identified through testing of infants born to women with primary CMV infection during pregnancy (*n* = 2), screening (*n* = 2), or testing for suspected infection or clinical indication (*n* = 4). One of two studies without a comparison group found adverse neurodevelopmental outcomes in 1/79 AcCMV,^19^ while the other reported mildly abnormal neurological evaluation in 6/54 AcCMV (but none with more severe abnormalities), and 14% (8/54) with motor neurodevelopmental delay at 6 months.^32^ Only 4/21 studies compared neurodevelopment in AcCMV and cCMV‐uninfected groups; one reported normal neurodevelopment in both groups^35^ while three reported a small and similar proportion of children with motor or neurodevelopmental delays in each group (Table 2).^24^, ^33^, ^34^

A further nine of the 21 studies reported some adverse neurodevelopmental outcomes among children with AcCMV and included comparisons with a ScCMV group.^20^, ^21^, ^22^, ^25^, ^26^, ^27^, ^29^, ^30^, ^38^ In seven of these nine studies, the prevalence of adverse outcomes was lower in children with AcCMV versus ScCMV^20^, ^21^, ^22^, ^26^, ^27^, ^29^, ^38^; for example, Blazques‐Gamero et al. reported neurological abnormalities in 1/20 AcCMV (none had SNHL) compared to 15/51 ScCMV (3 of which were in combination with SNHL).^20^ Koyana et al. reported findings from a cohort derived from newborn screening, where one case each of autistic spectrum disorder (ASD) and attention deficit hyperactivity disorder were reported among the 43 AcCMV along with 2 cases of speech delay, whereas adverse developmental and/or hearing outcomes were reported in 14/17 ScCMV.^27^ In the remaining two studies, Garofoli et al. reported similar proportions of children with ASD in the AcCMV and ScCMV groups (2/50 and 1/20 respectively).^25^ Maes et al. also found no significant difference in fine motor performance as measured by the Peabody development scale between 8 AcCMV, 8 ScCMV hearing‐impaired and 8 ScCMV children without hearing impairment. However, the ScCMV hearing‐impaired group presented lower gross motor performance compared to the AcCMV group overall, and within the ScCMV hearing impaired group, lower gross motor performance correlated with absent cVEMP (cervical vestibular evoked myogenic potential) responses indicating reduced vestibular function.^30^

### 3.3 Neurodevelopmental outcomes in studies with 5 years or more of follow‐up

Six studies (two from the same cohort) reported outcomes with a mean or median follow‐up or age at scheduled assessment of at least 5 years,^40^, ^41^, ^42^, ^43^, ^44^, ^45^ highlighted in red in Figure 2. Papaevangelou et al. reported normal psychomotor development, receptive and expressive language skills on the Griffiths mental development scale among five children with AcCMV and was the only study of the six not to include a CMV‐uninfected comparison group.^45^

Three papers reported on different outcomes from overlapping cohorts arising from a CMV screening study in the USA of children born in 1982–1992.^40^, ^41^, ^42^ No reports of IQ scores below 70 were reported among 76 children with AcCMV and 29 cCMV‐uninfected children by Topham et al. and there was no significant difference in attention problems or hyperactivity t‐scores ≥65 between the two groups.^42^ In an overlapping cohort, Lopez et al. compared combined IQ scores at later ages (up to 18 years) according to symptoms of cCMV at birth and hearing at age 2 years. They reported no difference in later combined IQ scores between 75 AcCMV who had normal hearing at age two and 39 CMV‐uninfected children; 11 children with AcCMV and SNHL diagnosed by two years of age scored lower on later measures of full‐scale intelligence and receptive vocabulary than the cCMV‐uninfected group but had similar scores across other measures.^41^ Finally, Jin et al. reported no difference in the proportion with neurodevelopmental delay between AcCMV and CMV‐uninfected children groups (4/109 and 1/51 respectively, Table 2), but adverse outcomes in 24/77 ScCMV.^40^

Korndewal et al. 2017a^43^ and 2017b^44^ reported clinician versus parent‐reported outcomes, respectively, from the same cohort. Of 133 children with cCMV in this study (107 AcCMV, of whom 3 had SNHL) and 274 matched cCMV‐uninfected children, medical records showed a higher prevalence of cognitive, motor and speech‐language impairment in the cCMV infected group (no difference for neurological impairment) (Table 2). On comparing the proportion with each impairment in the AcCMV and CMV‐uninfected groups using data from the paper, differences in neurological, cognitive or speech language impairment were not statistically significant, but children with AcCMV were more likely to have motor impairment (10.3% vs. 1.5% among cCMV‐uninfected, *p* < 0.01, no data on vestibular function).^43^ Parent‐reported measures showed a similar proportion of children with each delay between the cCMV‐infected group (AcCMV and ScCMV combined) and the cCMV‐uninfected, with the exception of expressive language (delays in 26.1% of ScCMV, 11% of AcCMV and 5.8% of uninfected) and general development (delays in 21.7% of ScCMV, 8% of AcCMV, 3.7% of uninfected). When we conducted statistical tests to compare the proportion with each delay in the AcCMV versus cCMV‐uninfected groups, the only one with weak evidence of an association was expressive language (*p* = 0.092) (all others, *p* > 0.1).^44^

### 3.4 Risk of bias assessment

Seven studies had no comparison group, therefore reducing the denominator for the scale from 7 to 5,^18^, ^19^, ^32^, ^36^, ^37^, ^39^, ^45^ as seen marked with ‘^a^’ in Table 2; of the remainder, most compared children with AcCMV and ScCMV, with only nine having a CMV‐uninfected comparison group.^24^, ^33^, ^34^, ^35^, ^40^, ^41^, ^42^, ^43^, ^44^ Frequent sources of bias were that selection of study populations was not representative (e.g., neonates were tested for cCMV due to symptoms), short follow‐up periods, and that study groups were difficult to compare due to a lack of information on other factors, for example, presence and degree of hearing loss.

## 4 DISCUSSION

In this review of 28 studies published in 2016–2022 reporting on neurodevelopmental outcomes of children with AcCMV, only nine compared outcomes between AcCMV and uninfected children. Six of these nine studies reported similar outcomes^24^, ^33^, ^34^, ^35^, ^40^, ^42^ (the two with follow‐up of at least 5 years were from overlapping cohorts), while three did report some differences, either limited to measures of full‐scale intelligence and receptive vocabulary among children with AcCMV and SNHL,^41^ or more generally in motor impairment and with borderline association for expressive language.^43^, ^44^ These latter two studies, published from the same retrospective cohort of six‐year‐olds in the Netherlands, did not report these statistical comparisons but reported data that allowed us to conduct these tests. Of note, overlap between hearing and motor impairment was not reported, but only three children with AcCMV had hearing loss while 11 had motor impairment; prevalence of hearing loss in these studies (collected from routine medical data) was lower than expected, and undiagnosed hearing loss or vestibular dysfunction could be linked with the motor and language delays observed among children with AcCMV.

Comparisons of neurodevelopmental outcomes between AcCMV and ScCMV were more widely reported (by 19 studies) and showed, as in previous literature,^46^, ^47^ that adverse outcomes were generally less common among children with AcCMV. The magnitude of this difference varied widely—likely in part driven by selection criteria for the included populations. Seven studies reported outcomes in children with AcCMV without any comparison group and were mostly small; five reported no adverse developmental outcomes,^18^, ^36^, ^37^, ^39^, ^45^ while two reported developmental delays in 1/79 and 8/54 children respectively with AcCMV.^19^, ^32^

The earlier review by Bartlett et al. included 20 studies published over a longer period (1974–2016) compared to our review (2016–2022) but with similar number of children with AcCMV with follow‐up overall (887 vs. 953, respectively). Six studies in each review had follow‐up to at least 5 years (representing 30% and 21% of included studies respectively), while 55% of studies in the earlier review versus 32% of studies in this review had a CMV‐uninfected comparison group. Isolated SNHL did not form part of the ScCMV definition in the earlier review, whereas the majority of studies included here (19/28) did include it in their ScCMV definition, reflecting introduction of newborn hearing screening over time. This may allow for exploration of other, related outcomes (such as speech and language development) in the AcCMV group in absence of hearing loss at birth, although a proportion are expected to develop hearing loss later.

As in this review, most studies in Bartlett et al.'s review did not show a difference in neurodevelopmental outcomes between AcCMV and cCMV‐uninfected groups. However, one cross‐sectional study showed poorer performance in full‐scale IQ and certain motor tasks among children with AcCMV aged 4–6 years, but not older ages,^48^ while another showed lower global development quotient (mainly impaired language development) in AcCMV compared to controls.^49^ Some of the main study limitations, such as short follow‐up times and lack of comparison groups, were similar across the reviews. In our review, although the retrospective cohort in the Netherlands had one of the stronger designs,^43^ there remained a risk of selection bias with only 44% of an initial 73,693 approached having consented to cCMV testing, and differential subsequent consent rates for data linkage among those with and without cCMV infection.

The variation in definitions used for ScCMV in studies included in this review (with 19 including isolated hearing loss, 17 including neuroimaging abnormalities) suggests that although the basis of current treatment guidelines,^50^ this categorisation may be less useful for clinical and research purposes more widely, and complicates comparisons between studies. Of note, children categorised as AcCMV received antivirals in 6/28 of the studies, probably reflecting variability in definitions of AcCMV. Development of more nuanced prognostic indicators will be important in informing groups to be included in future clinical trials, and clinical counselling of parents on longer term outcomes.

Only 12/28 studies reported on type (primary vs. non‐primary) or timing of maternal CMV infection,^21^, ^23^, ^24^, ^26^, ^28^, ^30^, ^31^, ^32^, ^33^, ^34^, ^35^, ^45^ reflecting the challenges in collecting this data and the design of many studies which were either retrospective or followed infants from birth. Almost all studies included in this review were conducted in high‐income countries, where a greater proportion of pregnant women than in low or middle‐income countries are at risk of a primary infection (averaging 30% in the European region).^51^ In a large population‐based prospective cohort study in Finland included in our review, 48% of children with cCMV were born to women with a primary CMV infection,^34^ similar to 52% from a previous French study,^52^ while this proportion was 4/15 and 1/10 in prospective cohorts in Spain and Greece respectively,^21^, ^45^ and none of the 123 children in a South Africa cohort with known maternal infection type.^33^ Although vertical transmission risk is much higher following maternal primary infection, the risk of adverse outcomes among infants with cCMV seems similar regardless of maternal infection type.^53^ In our review, four studies of children born to women with primary CMV infection showed generally quite small numbers of AcCMV with neurodevelopmental impairment at follow‐up (none,^35^ 2/19,^23^ 1/18,^24^ 8/54^32^) while a larger study stratifying outcomes by maternal infection type showed that all 6/70 AcCMV with neurodevelopmental impairment were born to mothers with primary maternal CMV infection.^26^ Although first trimester infection is important for risk of symptoms at birth,^54^ less is known about maternal timing of infection and later outcomes among children with AcCMV, and whether maternal serology is relevant to outcomes in this group.

### 4.1 Limitations

The interpretation of the included studies was limited by mostly short and highly variable follow‐up times, non‐representative samples of differing sizes (arising from different study designs—for example, populations screened for cCMV vs. those with clinical indications for testing), inconsistent definitions of AcCMV, and lack of an uninfected control group in many of the studies. Follow‐up times of less than 5 years, as in the majority of studies, will result in under‐ascertainment of relevant outcomes identified during school years and among groups more likely to experience diagnostic delays (e.g. girls with ASD^55^), and reduce statistical power to detect differences between groups. On the other hand, in prospective studies, children already diagnosed with cCMV may be more intensively monitored than cCMV‐uninfected controls, while loss to follow‐up can be more common among children with AcCMV than ScCMV^27^ and possibly associated with probability of later outcomes, introducing selection bias into study findings. The lack of cCMV‐uninfected comparison groups in the majority of studies limits conclusions about the extent to which adverse outcomes are attributed to cCMV, especially given that some of the outcomes explored are relatively common (e.g. ASD diagnosed in 1.76% of children in the general population in England overall by mean age 10.18 years^56^). In this review, the synthesis of results was also limited by the large variation in how neurodevelopmental impairment was defined and evaluated. Vestibular dysfunction can occur in children with cCMV both with and without hearing loss, and may contribute to motor delay^56^; however it is likely to be underdiagnosed, possibly particularly in children with AcCMV without hearing loss, and data on co‐occurring vestibular dysfunction was very limited in the studies included in this review.^57^ We only included studies published in English in this review, which is a limitation of our inclusion criteria. We also excluded grey literature, given that this is more likely to be low quality and difficult to interpret.

### 4.2 Implications for practice and policy—Explicit recommendations for future research

This review highlights the continued need for more robust, longer‐term data to understand neurodevelopment of children with AcCMV. In the UK, the annual cost of managing individuals of all ages with cCMV has been estimated at £732 million^58^ but this is based on an incomplete understanding of long‐term effects in infants without symptoms at birth, who may never be diagnosed despite experiencing sequelae. A better understanding of the long‐term neurodevelopmental outcomes of AcCMV has wide‐ranging implications including for informing screening policy, the cost‐effectiveness of future vaccine implementation (with a phase 3 CMV vaccine trial by Moderna currently underway^59^), and inclusion of cCMV testing into protocols for investigation of neurodevelopmental delay/disorder, which requires availability of routine DBS samples from the newborn period. Inconsistencies in outcome measures and lack of statistical power can be addressed through shared protocols and multi‐centre studies, as already shown in the Spanish studies.^20^, ^21^ The Spanish database is now the basis for the European Registry, which already has more than 1050 children included.^60^ However, observational studies of diagnosed children will continue to focus on infants with hearing loss at birth and the ScCMV group. Universal neonatal screening for cCMV has been recently introduced in Minnesota, Ontario and Saskatchewan,^61^ creating an opportunity for collection of outcome data irrespective of maternal diagnosis or neonatal symptoms. Study designs are needed that focus on clearly defined neonatal and longer‐term outcome measures of children with AcCMV, including degrees and timing of hearing loss, rehabilitation, and vestibular function measures alongside neurodevelopment. A better understanding of prognostic neonatal biomarkers (whether brain imaging, transcriptomic patterns, or others), as well as robust neurodevelopmental measures are required to fully understand the true spectrum of the longer‐term effects of AcCMV.

In conclusion, there remains little current evidence indicating that children with AcCMV are at higher risk of experiencing neurodevelopmental sequelae compared to cCMV‐uninfected controls from the 28 studies included in this review. However, this conclusion is limited by differing neurodevelopment assessment methods, short follow‐up times, a lack of comparison groups and possible bias in study inclusion and/or outcome ascertainment.

## AUTHOR CONTRIBUTIONS

**Angeliki Smyrli**: Conceptualization; methodology; investigation; writing – original draft. **Vishnuga Raveendran**: Conceptualization; methodology; investigation; writing – original draft; visualization. **Simone Walter**: Methodology; writing – review & editing. **Waheeda Pagarkar**: Methodology; writing – review & editing. **Nigel Field**: Methodology; writing – review & editing. **Seilesh Kadambari**: Methodology; writing – review & editing. **Hermione Lyall**: Methodology; writing – review & editing. **Heather Bailey**: Conceptualization; methodology; investigation; writing – original draft; supervision; project administration; funding acquisition.

## CONFLICT OF INTEREST STATEMENT

Hermione Lyall is the chair of CCMVNET. The other authors have no conflicts to disclose.

## ETHICS STATEMENT

N/A for this systematic literature review.

## PATIENT CONSENT

N/A for this systematic literature review.

## PERMISSION TO REPRODUCE MATERIAL FROM OTHER SOURCES

N/A.

## Data Availability

Data sharing is not applicable to this article as no new data were created or analysed in this study.
